# Cost-effectiveness of talazoparib for patients with locally advanced or metastasized breast cancer in Germany

**DOI:** 10.1371/journal.pone.0278460

**Published:** 2022-12-01

**Authors:** Florian Schwarz, Habibollah Arefian, Michael Hartmann, Ingo Runnebaum

**Affiliations:** 1 University Hospital Jena, Jena, Germany; 2 German Cancer Research Center, Heidelberg, Germany; 3 Hospital Pharmacy, Jena University Hospital, Jena, Germany; 4 Cancer Center Central Germany, Berlin, Germany; 5 Clinic for Gynecology and Reproductive Medicine, Jena University Hospital, Jena, Germany; Nnamdi Azikiwe University, NIGERIA

## Abstract

This study evaluated factors that influence the cost-effectiveness of talazoparib, particularly for patients with a germline breast-cancer-gene-(brca)-mutation and locally advanced or metastasized breast cancer within the context of the German healthcare system. We constructed a partitioned survival model to compare medical costs and treatment effectiveness for patients with such cancers over 45 months. Transition probabilities were derived from survival data from a randomized Phase-III EMBRACA trial, utilities based on published reports, and costs in Euros, which included costs for drug acquisition, clinical monitoring, and treatment of adverse events. Willingness-to-pay thresholds were set to be multiples of the current German per capita gross domestic product. Treatment with talazoparib led to a gain of 0.32 life-years (0.22 quality-adjusted life-years). The mean total cost of €84,003 for talazoparib and €12,741 for standard therapy resulted in an incremental cost-effectiveness ratio of €223,246 per life-year and €323,932 per quality-adjusted life-year gained, indicating that talazoparib is unlikely to be cost-effective at current pricing.

## Introduction

Nearly 70,000 new cases of breast cancer arise in Germany every year, meaning that one in eight women will develop this cancer type during her lifetime [1]. Despite great improvements in therapy options over the last decade, mortality remains high and breast cancer accounts for the greatest proportion of cancer deaths in women [2].

Targeted therapies such as poly(adp-ribose)-polymerase-inhibitors (PARPis) offer new therapeutic options for different cancer types [3,4]. This relatively new family of drugs can be used to treat breast-cancer-gene-(BRCA)-positive cancer due to their exploitation of insufficient DNA-repair in BRCA-positive cells and their ability to trap PARP [5,6]. The phase III EMBRACA trial found talazoparib to be superior to currently available drugs regarding median disease progression-free survival (8.6 months [95% CI: 7.2–9.3] vs. 5.6 months [CI: 4.2–6.7) [7], delay in time to definitive clinically meaningful deterioration and overall improvement in quality of life (QoL) [8]. Clinically meaningful deterioration was defined as a ≥10-point increase with no subsequent <10-point decrease from baseline ratings on the Global Health status/ Quality of Life (GHS/QoL) questionnaire [8]. These results remained stable among clinically relevant subgroups, including those based on age, BRCA-status and prior treatment [9]. During the trial, the new drug showed a favorable and consistent adverse effect profile [10]. Talazoparib is the fourth PARP-inhibitor to be approved and marketed, following olaparib, niraparib and rucaparib, and the second to be approved for breast-cancer treatment by the US Food and Drug administration (FDA) [11]. The drug is also approved by the European Medicines Agency (EMA) and the German federal Institute for Pharmaceuticals and Medical Products (Bundesinstitut für Arzneimittel und Medizinprodukte, BfArM). On November 20, 2020, the German federal joint committee (Gemeinsamer Bundesausschuss, GBA) assigned a considerable additional benefit to talazoparib in patients with previously treated, late-stage breast cancer [12,13]. A more effective new treatment for breast cancer could have a substantial impact on German health care expenditure. In 2018 about €7.0 billion of a total €42 billion German drug expenditure was for treatment of cancer, making oncologic drugs the most expensive drug group though accounting for only a relatively small percentage of total prescriptions (7.1 out of 285.3 million in 2018) [14]. Among all cancer-types breast cancer is associated with the highest health care costs [15], accounted for most prominently by newly approved treatments including talazoparib [14].

In recent years, the high cost of newly approved drugs has raised concerns regarding impact on the social health system [14,16–18]. As resources are limited, proof of evidence of a new drug´s effectiveness is essential to assure efficient spending. However, a recent article found over half the drugs released between 2011 and 2017 lacked evidence of benefit [19]. For PARP-inhibitors, there are some cost-effectiveness-analyses from the American health-care system [20–23] as well as a recent Spanish study of talazoparib [24]. However, to our knowledge the present study is the first to focus on the German market, where data are still lacking.

The objective of this study was to explore the influence of different factors on the cost-effectiveness of talazoparib and to examine under which circumstances the new drug might prove cost-effective within the context of the German healthcare system.

## Materials and methods

We constructed a partitioned survival model using TreeAge Pro 2019 software (TreeAge Pro 2019, R1.1; TreeAge Software, LLC, Williamstown, MA, USA). Data were obtained from published reports of a randomized, phase-3 EMBRACA [7,8,10]. The patient population was described previously in the protocol of the EMBRACA trial [7]. The trial included 431 patients from several countries including Germany and assigned them in a 2:1 ratio to either receive talazoparib (1mg once daily) or standard single-agent chemotherapy (capecitabine, vinorelbine, gemcitabine or eribuline) in continuous 21-day cycles. Platinum-based therapies were not included among standard therapies [7]. 82.9% of patients in the talazoparib and 84.0% in the control group had received previous adjuvant or neoadjuvant therapy [25].

### Model structure

**Fig 1:**
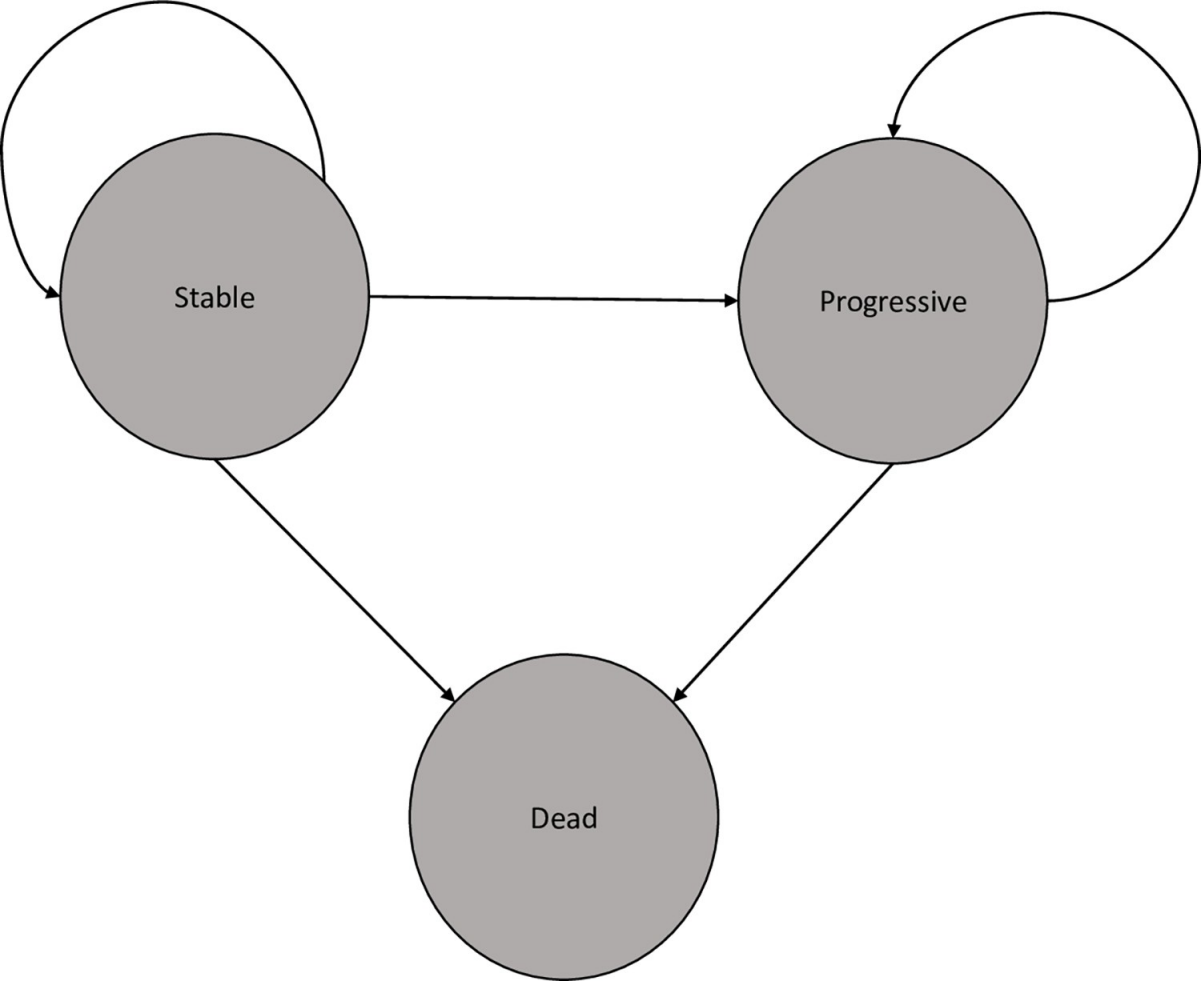
Model structure.

Our model compared the costs and effectiveness of treatment based on life years (LY) and quality-adjusted life-years (QALY) gained with standard treatments vs. with talazoparib. The model includes three health states, reflecting the natural process of the disease: stable, progressive and dead. All patients started in the stable state, where they could remain, move on to the progressive state, or die. Transition to the progressive state defined disease progression in the EMBRACA trial [7]. Backward changes from progressive to stable state were not permitted. The structure of our model is presented in Fig 1.

We used time-dependent transition probabilities as they are more convenient in modeling progressive diseases than constant values [26,27]. Transition probabilities were based on Kaplan-Meier survival functions from the EMBRACA trial and were not influenced by the patient´s medical history [26].

The median overall survival in the EMBRACA trial was 22.3 months (CI: 18.1–26.2) with talazoparib and 19.5 months (16.3–22.4) with standard treatments. The median follow-up for PFS was 11.2 months in the EMBRACA trial [7]. We set the time horizon at 45 months, or 5 months longer than the maximum survival registered in the EMBRACA trial in which the 40-month survival rate was 0% with talazoparib [7,24]. This was done to include all survival data points available and allow for a sufficient estimation of all costs and health outcomes. Having the time horizon longer than the reported follow-up made extrapolation necessary.

As suggested by Ishak et al. we used the method of minimizing squared residuals and fitted five parametric distributions to Kaplan-Meier curves of overall survival (OS) and progression free survival (PFS) [28], based on exponential, log-normal, log-logistic, Weibull and Gompertz methods. In each case the best-fitting curve was identified by the sum of squared residuals, the Akaike information criterion and the Bayesian information criterion, as well as discussion with University of Jena oncologists, following the recommendations of Latimer [29]. We selected a log-normal function for the talazoparib group and a Gompertz function for the standard therapy comparison group.

Transition probabilities were calculated directly from Kaplan-Meier-data of the EMBRACA trial or, if where no data were available, from the extrapolations, using the formula:

$${P=1-e}^{\frac{lny}{t}}$$

Where *P* is the transition probability and *y* is the survival rate (from Kaplan-Meier curve or extrapolated) at a given time *t* [26]. The transition probabilities for “stable to progressive” and “stable to dead” were calculated using yPFS and yOS, respectively. The transition probability from progressive disease to death was calculated from the difference between yOS and yPFS. Its decrease is caused by steady decline of both OS and PFS curves over time. To confirm that the results were caused not only by the choice of the survival curve we ran confirmatory analyses in which survival curves of both treatment arms were based on the same function. Transition probabilities and calculations are provided in the supplementary Appendix.

A 30-day treatment cycle-length was selected. Following recommendations from the German Independent Institute for Quality and Efficiency in Healthcare (Institut für Qualität und Wirtschaftlichkeit im Gesundheitswesen, IQWIG) we applied a yearly discount of 3% to costs and utilities [30].

### Utilities

The efficacy of new chemotherapeutic drugs is affected not only by their capability of prolonging life, but also by their ability to support a high QoL for as long as possible. Choosing appropriate utilities is therefore a crucial step in constructing a partitioned survival model. Utility values for breast cancer are inconsistent and vary greatly among studies [31,32]. The EMBRACA-trial assessed QoL using the European Organization for Research and Treatment of Cancer Quality of Life Questionnaire Core 30 (EORTC QLQ-30) and its breast cancer module, QLQ-BR23 [8], scores for which range from 0 (worst) to 100 (best) [33]. Mean baseline scores were similar in both treatment groups (61.9 within talazoparib and 60.9 within standard treatment).

However, as utility values were not explicitly reported, we had to seek published values, using the Pubmed database with the key words “utility values,” “breast cancer,” and “metastasized”. This search yielded 60 results, which we screened manually. They included a review by Paracha et al., who summarized health-state utility values for the population in question while adhering to high standards [32]. They screened over 2000 publications to find health-state utility values for locally advanced and metastatic breast cancer and found 24 reports with original utility values, nine of which met the standards of health technology assessment agencies [32]. We decided on 0.66 for the stable and 0.44 for the progressive state as reported by Sherril et al. [34] regarding a phase III randomized trial which compared capecitabine plus lapatinib with capecitabine monotherapy [35]. The decision was supported by the relatively large population size involved and its similarity to the patient sample of the EMBRACA trial. These values also were used in a Spanish cost-effectiveness analysis on talazoparib [24]. We acknowledge that our values are not ideal as they are not based upon the profile of talazoparib or another PARP inhibitor; however we tried to cover the effect of having different utility values for each treatment arm through several comparison analyses.

### Cost calculation

#### General

We included only direct medical costs, consisting of those for drug acquisition, hospitalization, monitoring, and treatment for adverse events. Adopting the perspective of German health insurance we matched health items with the German diagnosis related groups (DRG) system for hospitalization and the German uniform value scale catalogue (Einheitlicher Bewertungsmaßstab, EBM) for outpatient procedures [36]. DRG-values were estimated with a reimbursement information application of 2019 [37]. Drug prices were deduced from the pharmacy database Lauer-Taxe of December 2020. Monthly costs are summarized in in Table 1.

**Table 1:** Cost summary.

	Proportion of treatment arm				Monthly Costs (€)	
Talazoparib					6810.8	
Standard chemotherapy (including gemcitabine)					1363.4	
Standard chemotherapy (excluding gemcitabine)					1173.2	
Capecitabine	0.4				211.4	
Gemcitabine	0.1				759.4	
Vinorelbine	0.1				935.4	
Eribuline	0.4				2822.3	
Surcharge for preparation of intravenous treatment					115.7	
Monitoring						
Consultation					27.4	
Laboratory					19.1	
Imaging					75.3	
AEs	Incidence (total, %)	Treatment cost (inpatient, €)	Treatment cost (outpatient, €)	Hospitalization (€)	Cost (first month, €)*	Cost (second month and following, €)*
Diarrhea (talazoparib)	0.7	1.7	0.4	1162.8	8.1	0.0
Anemia (talazoparib)	39.2	1837.5^a^	1468.1	1056.4	428.9	16.0
Neutropenia (talazoparib)	10.1	1811.3^b^	91.3	953.6	96.5	4.1
Thrombozytopenia (talazoparib)	3.5	437.0	437.0	953.6	33.7	0.3
Total AE treatment cost (talazoparib)					567.2	20.4
Diarrhea (standard therapy)	5.6	1.7	0.4	1162.8	65.1	0.0
Anemia (standard therapy)	4.8	1766.5^a^	1397.1	1056.4	75.0	1.9
Neutropenia (standard therapy)	17.5	2068.2^b^	104.2	953.6	167.3	8.0
Total AE treatment cost (standard therapy)					307.4	9.9

[*] incidence adjusted; a. use of packaged red blood cells differed between talazoparib and standard therapy (median, 2 vs 1) [10]; b. growth factor use differed between talazoparib and standard therapy (10.1% vs 17.5%). Drug use is based on the protocol of the EMBRACA trial as well as German guidelines and medication package inserts; *AE =* adverse event.

#### Costs of drug acquisition

The frequency of drug administration followed the EMBRACA study protocol [7]. If there were different package sizes available, we chose the least costly.

Talazoparib is taken orally once daily; its price for one package with 30 1-mg tablets was €6810.82 in December 2020.

Treatment regimens differed among the four drugs used for standard therapy. These drugs were administered either orally (capecitabine) or by intravenous infusion (vinorelbine, gemcitabine and eribuline) on specific days in 21-day-cycles. For a better comparison with talazoparib treatment we calculated an average price per 30 days based on the representation of each drug in the control group. To determine drug dose, we assumed an average body surface area of 1.7 m^2^, as representative of German patients [38].

According to the EMBRACA trial protocol, treatment was to be continued until disease progression, unacceptable toxic effects, withdrawal of consent or physician decision led to stopping. Adverse events resulting in discontinuation occurred in 5.9% of the 286 patients in the talazoparib and 7.6% of the 144 patients in the standard treatment group. Dose modifications due to adverse events occurred in 66% of patients given talazoparib and 60% of those given standard treatment [7]. Some information was available on the incidence of dose-modification with talazoparib, but not with the standard treatment [7]. Information on dose modifications in the control arm was not available. Therefore, we included only full drug costs associated with both treatments.

#### Costs of adverse event treatment

After discussion with oncologists at Jena University Hospital, we included only adverse events of stage III or IV severity, based on the Common terminology of Adverse Events-(CTCAE)-scale [39]. We then used a contingency table (c^2^) to select for adverse events that showed significant difference (p<0.05) between the treatment groups. The standard treatments were chosen based on German guidelines [40] supplemented with expert opinion. The duration of treatments was based on EMBRACA trial data as well as medication package inserts. As monthly or yearly rates of adverse events were not available, we calculated their monthly cost by multiplying the cumulative incidences reported from the EMBRACA trial [7] by adverse event treatment cost and dividing by the time horizon. We matched adverse events requiring hospital treatment to an adequate DRG and assumed a one-day-long hospitalization per afflicted patient for our base-case analysis. These adverse-event-induced hospitalization costs were included in the first cycle of the stable state.

#### Costs of disease monitoring

Costs of disease monitoring include consultation, laboratory, and imaging costs. For consultation we assumed one per quarter per case. The laboratory panel as well the monitoring schedule were deduced from the EMBRACA trial study protocol and a monthly cost was calculated based on the EBM [7,36]. The EMBRACA trial protocol included brain scan (CT or MRI) every 6 weeks for the first 30 weeks and every 9 weeks thereafter, as well as a bone scan every 12 weeks, all in accordance with German guidelines on metastasized breast cancer [40].

#### Willingness-to-pay (WTP)

The German IQWIG uses an approach that differs from many other countries as it bases decisions on an “efficiency limit” (“Effizienzgrenze”) rather than on fixed values. The efficiency limit is based on available treatments and can vary with the indications present [30]. This unique approach makes the results difficult to interpret and to apply to international comparisons. We therefore decided to use fixed thresholds based on gross domestic product (GDP) per capita to improve international comparability and selected three-times the GDP per capita as a primary threshold.

### Analysis

#### Primary outcome

The objective of this study was to explore the influence of different factors on the cost-effectiveness of talazoparib and to examine under which circumstances the new drug might be cost-effective for patients with a germline brca-mutation and locally advanced or metastasized breast cancer within the context of the German healthcare system. Cost-effectiveness was reflected in the difference of the incremental cost and utility (incremental cost effectiveness ratio, ICER). The ICER was quantified in € per LY and € per QALY. Cost-effectiveness thresholds were assumed to be multiples of GDP per capita.

#### Scenario and probabilistic analyses

For the scenario analyses we applied 50% variation on drug and adverse effect costs and 25% on monitoring costs to determine their influence on the ICER. We chose different variation values because in Germany drug costs are usually subject to higher uncertainty than established monitoring procedures, for example due to the AMNOG (Arzneimittelmarkt-Neuordnungsgesetz, the Reform of the Market for Medicinal Products Act) or discounting contracts between payers and producers, while latter tend to remain relatively stable over time [36]. As we selected utility values from the literature and had no other information concerning the uncertainty of these estimates, we assumed 20% variation for utility variables. For discounting we used border values of 0% and 5% as recommended by IQWIG [30].

For probabilistic analyses major model inputs were varied simultaneously in 10,000 iterations to explore the likelihood of talazoparib being cost-effective. Following the recommendations of IQWIG we used a gamma-distribution for costs. Confidence intervals of utilities were estimated by a normal distribution which included values between 0 and 1.

We also applied a threshold analysis to find the price at which talazoparib would prove cost-effective. This was done by varying the pricing variable while keeping all other variables constant.

#### Utility analyses

Findings from the EMBRACA trial suggest an improved quality of life with talazoparib compared to standard therapy [7,8]. We included a bivariate analysis to explore the effect of higher utility values with talazoparib treatment. Talazoparib utilities were varied from 80% to 120%. Furthermore Ettl et al. found time to definitive clinically meaningful deterioration significantly delayed among talazoparib treated subjects (24.3 [13.8–infinity] vs 6.3 months [4.9–14.2]) [8]. Within chronic diseases health usually deteriorates over time. We therefore included two scenario analyses with a steady utility decline, subtracting a certain amount of utility with each cycle (-0.005 utility points for slow and -0.01 for fast decline). This is sometimes done to reflect health deterioration in chronic diseases like breast cancer [41]. Scenarios and utilities are depicted in Table 4.

## Results

### Base case results

Therapy with talazoparib led to a gain of 0.32 LYs more than with established treatments. Adjusted for QoL, talazoparib treatment resulted in an additional 0.22 QALYs. The mean total cost was €84,003.3 within talazoparib and €12,741.0 with standard treatments. The ICERs for talazoparib vs. standard treatments were €323,931.6 per QALY and €223,246.0 per LY. Table 3 shows a cost and utility breakdown for the base case. The results of the confirmatory analyses in which survival curves of both treatment arms were based on the same function showed only minor deviations from our base case (maximum as much as 0.5 quality-adjusted life months) and can be found in the supplementary Appendix.

Talazoparib drug cost was the biggest cost-driver and accounted for over 90% of the cost in the talazoparib group. In the standard therapy group drug cost also accounted for the biggest proportion of the total cost. Costs for monitoring and AE treatment only had minor impact on total costs. The disaggregated outcomes are displayed in Table 2.

**Table 2:** Disaggregated outcomes (costs and utilities).

	Talazoparib	Standard therapy	
	Costs in € (%)	Costs in € (%)	Incremental cost (%)
Talazoparib	80486.7 (95.8)	0 (0)	80486.7 (98.8)
Standard Therapy	0 (0)	10196.7 (80.0)	
AE	972.5 (1.2)	466.7 (3.7)	505.8 (0.6)
Consultation	572.9 (0.7)	467.9 (3.7)	105.1 (0.1)
Lab	397.9 (0.5)	324.9 (2.6)	73.0 (0.1)
Imaging	1573.2 (1.9)	1284.7 (10.1)	288.5 (0.4)
Total	84003.3 (100.0)	12741.0 (100.0)	81459.1 (100.0)
	LY/QALY	LY/QALY	Incremental LY/QALY
Stable LY	0.98	0.62	0.36
Progressive LY	0.76	0.8	-0.04
Total LY	1.74	1.42	0.32
Stable QALY	0.65	0.41	0.24
Progressive QALY	0.33	0.35	-0.02
Total QALY	0.98	0.76	0.22

*AEs* = adverse events, *LY =* life year, *QALY* = quality-adjusted life year. Total numbers may be affected by rounding errors.

### Scenario analyses

**Fig 2:**
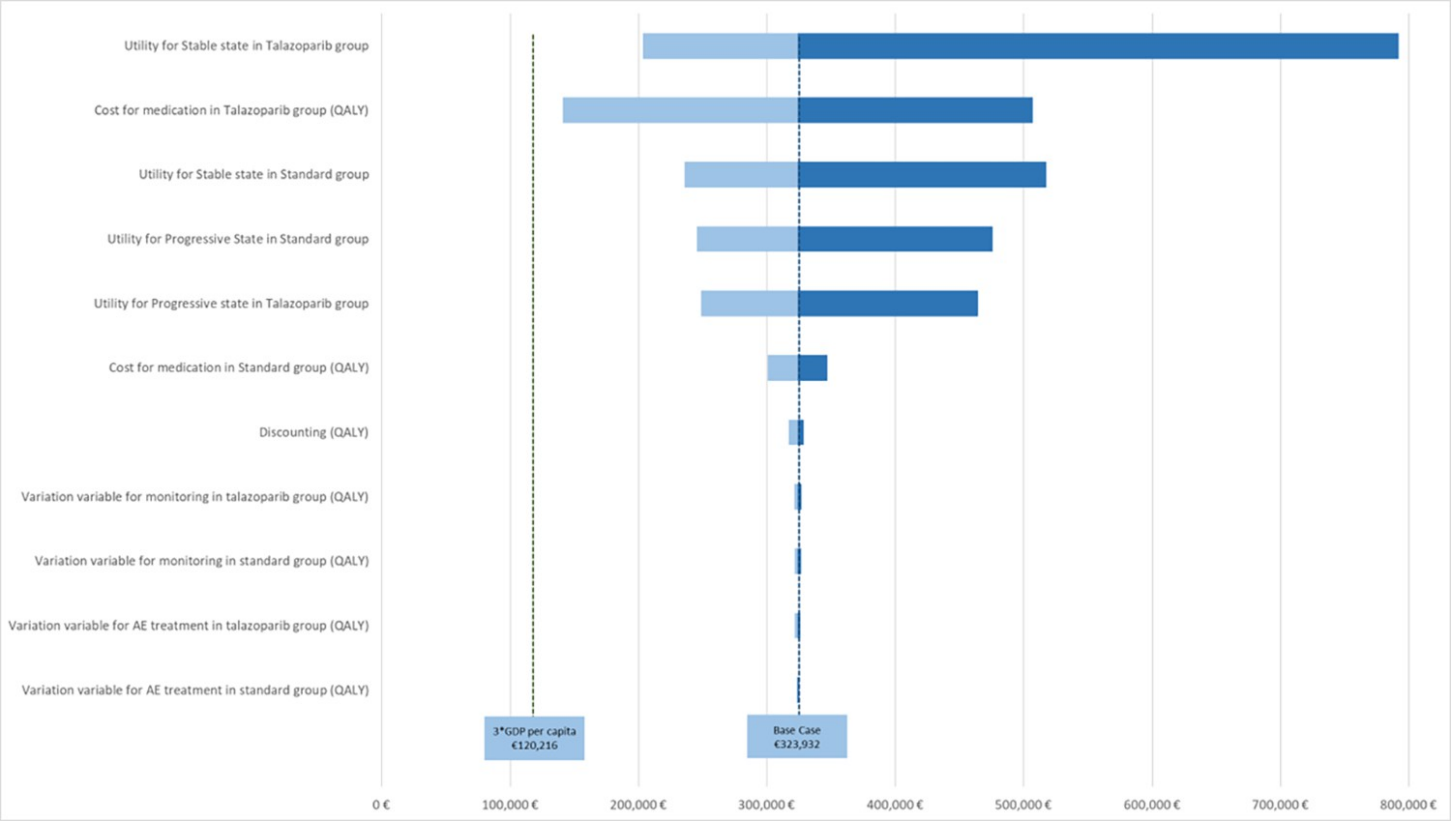
Scenario analysis. *QALY =* quality-adjusted life year, *AE =* adverse event, *GDP =* gross domestic product.

The results of our scenario and threshold analyses are shown in Fig 2. By threshold analysis, our model found the monthly price of talazoparib (30 days requires 1 package at regular dosing) would have to be reduced to about €3018.7 to be considered cost-effective at a WTP-threshold of 3 times GDP per capita or €1562.7 at a threshold of 1 times GDP per capita.

The ICERs of multiple testing scenarios regarding QoL are shown in Table 3. Unsurprisingly the increase of utility in both living states of the talazoparib group increased cost-effectiveness. Having both utilities at 120% of their original value led to an incremental effectiveness of 0.42 QALY and an ICER of €171,099.4, which is still above the 3 times GDP per capita /QALY threshold. Assuming a two-times faster utility decline in the standard therapy group also made the treatment more cost-effective, but reaching the cost-effectiveness threshold was still unlikely.

**Table 3:** Utility scenario analysis.

Scenario	Utilities				Incremental Effectiveness (QALY)	ICER (€)
	Stable		Progressive			
	Talazoparib	Standard	Talazoparib	Standard		
Basecase	0.66		0.44		0.22	323,931.6
BVA	0.792	0.66	0.44	0.44	0.35	203,615.9
BVA	0.66	0.66	0.528	0.44	0.29	248,730.3
BVA	0.792	0.66	0.528	0.44	0.42	171,099.4
Slow Decline in both groups	0.66		-0.005 per cycle	-0.005 per cycle	0.21	337,505.5
Slow decline in TLZ, faster with ST	0.66		-0.005 per cycle	-0.01 per cycle	0.29	247,892.1

*BVA =* bivariate analysis; *ICER =* incremental cost-effectiveness ratio (QALY); *ST* = standard therapy; *TLZ* = talazoparib; *QALY =* quality-adjusted life year.

**Fig 3:**
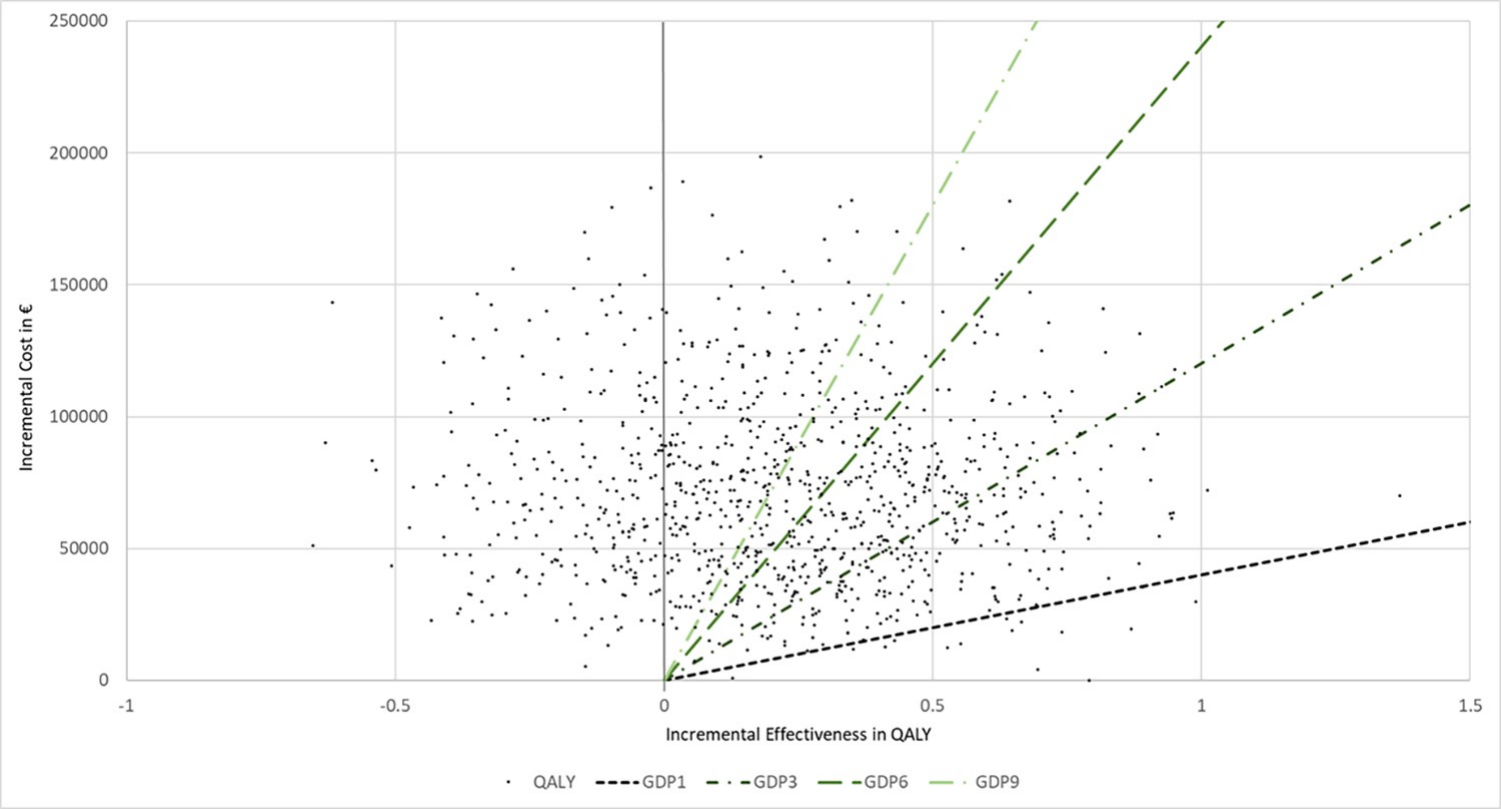
ICER scatterplot with willingness-to-pay thresholds according to GDP per capita. *QALY =* quality-adjusted life year; *GDP3 =* gross domestic product times 3.

Results of the probabilistic sensitivity analysis are shown in Fig 3. Assuming a WTP of 3 times the GDP per capita/QALY, talazoparib had a probability of being cost-effective of only about 19%. The probabilities of achieving cost-effectiveness at the chosen thresholds are provided in Table 4.

**Table 4:** Probability of achieving cost-effectiveness at estimated willingness-to-pay thresholds.

Thresholds based on GDP per capita in € (2020)		Probability of achieving cost-effectiveness threshold in % (QALY)
Factor	Cost	
1	40072	2.3
3	120216	18.9
6	240432	40.7
9	360648	52.1

*GDP* = gross domestic product; *QALY =* quality-adjusted life year.

## Discussion

In our base case analysis talazoparib proved to be more effective than standard therapy, with a gain of 0.22 QALYs or 0.32 LYs. Based on the digitized survival curves from the EMBRACA-trial [7], we calculated a mean survival gain of 2.6 months. Due to censoring, the actual value is likely to be slightly higher, which corresponds to our results. However, at an assumed WTP-threshold of 3 times GDP-per-capita /QALY (and per capita/LY) talazoparib was not cost-effective compared to established treatments. The main cost-driver was the price of talazoparib, which accounted for over 90% of all costs. Cost-effectiveness studies of other PARP-inhibitors came to similar conclusions, finding the new drugs more effective than placebo or standard therapy, but usually not cost-effective [20,23]. High costs for novel treatments are not a PARPi-specific issue but affect other drug-classes as well [42,43] and have been subject of criticism [17,44]. Costs found by Olry de Labry et al. for treatment with talazoparib were lower than in our present findings, but they assumed lower drug prices [24].

### Study limitations

The present study has considerable limitations. As the EMBRACA trial was the first study on talazoparib allowing for cost-effectiveness analysis, we relied solely on this source. Due to different guidelines in Germany and the US, relying only on the study protocol might lead to uncertainty, which we tried to mitigate through information from German guidelines and expert opinion. Secondly, as we had to rely solely on available OS and PFS data we calculated the transition probability from stable to dead from OS. This is not ideal, as OS can also include patients who died while in a progressive state. This circumstance could lead to a higher transition probability for stable-to-dead and to underestimating costs and health benefits.

Moreover, we did not include the effect of dose reduction or interruption in our model, as data on the amount and duration of dose reduction or interruption, especially regarding the standard therapy arm, were unavailable. Using a smaller dosage or interrupting the therapy will lead to a lower drug consumption and lower costs. In the EMBRACA trial 66% of patients in the talazoparib arm and 60% randomized to standard treatment experienced dose-modification at some point during the trial [7]. Therefore, our basecase, which assumes that all patients receive a full dose over the course of treatment, might overestimate the costs. The possibility of lower drug costs is covered in our scenario analyses (Table 3) where even with a broad variation of 50% the cost-effectiveness threshold could not be reached.

Another limitation is the lack of suitable utility data. As we could not find suitable data on talazoparib we had to use utility values from the literature [32,34]. This decision was based on the comparability to the patient cohort from the EMBRACA trial and the fair sample size. The EMBRACA trial found that patients given talazoparib had an improved QoL [7,10] and that QoL deteriorated more slowly with talazoparib group than with standard treatment [8]. Based on these findings, it is possible that the real utility values for talazoparib might be higher in later treatment cycles and the new drug´s effectiveness might be underestimated. A possible approach would have been to assign disutility in the occurrence of adverse events but considering that monthly adverse event incidence rates were not available, we decided against it. We instead ran multiple scenario analyses assuming different base values and a utility decline, none of which found talazoparib to be cost-effective.

Other authors have illustrated an issue concerning late-stage treatments for cancer that is common within this kind of models including the present study [22]. Most costs are accumulated during the stable state, meaning the longer a patient´s health does not deteriorate, the higher the cost. Yet in late stages of a chronic disease like breast cancer, the utilities can be reduced significantly in the stable state, leading to worsened cost-effectiveness and to undermining of a new drug´s benefit. This issue is difficult to address as it stems from the structural assumptions of the model. However, in our utility scenario analysis we included the possibility of higher utility values in the stable state to mitigate this effect.

In Germany, talazoparib is currently approved only for locally advanced or metastasized brca-positive breast cancer that has been treated previously with an anthracycline or a taxane and so is used mainly in late stages of disease. Administrating talazoparib in earlier treatment phases may improve the health benefits and cost-effectiveness obtained with talazoparib. This is subject of current investigation and a recent pilot study found talazoparib monotherapy to be effective for neoadjuvant treatment in BRCA-positive breast-cancer [45].

Taking everything into account, the pricing of talazoparib is the main cost-driver in its use and had a significant adverse impact on the cost-effectiveness. Finding an appropriate price for a new treatment remains a challenge for all stakeholders to allow for both efficient resource management as well as reward for innovation. The lack of valid cost-effectiveness data before approval has been criticized and underlines the need for post-approval assessments to allow for pricing adjustments [19].

## Conclusions

In conclusion, talazoparib is likely to be more effective than current standard treatment for breast cancer. However, at its current price it is not cost-effective. A higher probability for cost-effectiveness can be achieved by significant reduction of the acquisition price of talazoparib.

## Supporting information

S1 Data (XLSX)

## References

[1] Krebs in Deutschland für 2015 und 2016: Krebs in Deutschland für 2015/2016. 12. Ausgabe. Robert Koch-Institut (Hrsg) und die Gesellschaft der epidemiologischen Krebsregister in Deutschland e.V. (Hrsg). Berlin, 2019; 2019 [11.07.2020]. Available from: https://www.krebsdaten.de/Krebs/DE/Content/Publikationen/Krebs_in_Deutschland/kid_2019/krebs_in_deutschland_2019.pdf?__blob=publicationFile.

[2] WHO and International Agency for Research on Cancer (IRAC): Cancer today. [13.06.2021]. Available from: https://gco.iarc.fr/today/online-analysis-multi-bars?v=2018&mode=cancer&mode_population=countries&population=900&populations=900&key=asr&sex=2&cancer=39&type=0&statistic=5&prevalence=0&population_group=0&ages_group%5B%5D=0&ages_group%5B%5D=17&nb_items=10&group_cancer=1&include_nmsc=1&include_nmsc_other=1&type_multiple=%257B%2522inc%2522%253Atrue%252C%2522mort%2522%253Atrue%252C%2522prev%2522%253Afalse%257D&orientation=horizontal&type_sort=0&type_nb_items=%257B%2522top%2522%253Atrue%252C%2522bottom%2522%253Afalse%257D&population_group_globocan_id.

[3] Harbeck N, Gnant M. Breast cancer. Lancet. 2017;389(10074):1134–50. Epub 2016/11/21. doi: 10.1016/S0140-6736(16)31891-8 . 27865536

[4] Shin SH, Bode AM, Dong Z. Precision medicine: the foundation of future cancer therapeutics. NPJ Precision Oncology. 2017;1(1):12. Epub 2017/04/24. doi: 10.1038/s41698-017-0016-z ; PubMed Central PMCID: PMC5871793. 29872700 PMC5871793

[5] Helleday T. The underlying mechanism for the PARP and BRCA synthetic lethality: clearing up the misunderstandings. Molecular Oncology. 2011;5(4):387–93. Epub 2011/08/09. doi: 10.1016/j.molonc.2011.07.001 ; PubMed Central PMCID: PMC5528309. 21821475 PMC5528309

[6] Murai J, Huang SY, Renaud A, Zhang Y, Ji J, Takeda S, et al. Stereospecific PARP trapping by BMN 673 and comparison with olaparib and rucaparib. Molecular cancer therapeutics. 2014;13(2):433–43. Epub 2013/12/21. doi: 10.1158/1535-7163.MCT-13-0803 ; PubMed Central PMCID: PMC3946062. 24356813 PMC3946062

[7] Litton JK, Rugo HS, Ettl J, Hurvitz SA, Goncalves A, Lee KH, et al. Talazoparib in Patients with Advanced Breast Cancer and a Germline BRCA Mutation. The New England Journal of Medicine. 2018;379(8):753–63. Epub 2018/08/16. doi: 10.1056/NEJMoa1802905 . 30110579 PMC10600918

[8] Ettl J, Quek RGW, Lee KH, Rugo HS, Hurvitz S, Goncalves A, et al. Quality of life with talazoparib versus physician’s choice of chemotherapy in patients with advanced breast cancer and germline BRCA1/2 mutation: patient-reported outcomes from the EMBRACA phase III trial. Annals of Oncology. 2018;29(9):1939–47. Epub 2018/08/21. doi: 10.1093/annonc/mdy257 . 30124753

[9] Rugo HS, Ettl J, Hurvitz SA, Gonçalves A, Lee KH, Fehrenbacher L, et al. Outcomes in Clinically Relevant Patient Subgroups From the EMBRACA Study: Talazoparib vs Physician’s Choice Standard-of-Care Chemotherapy. JNCI cancer spectrum. 2020;4(1):pkz085. Epub 2020/04/28. doi: 10.1093/jncics/pkz085 ; PubMed Central PMCID: PMC7050154. 32337496 PMC7050154

[10] Hurvitz SA, Goncalves A, Rugo HS, Lee KH, Fehrenbacher L, Mina LA, et al. Talazoparib in Patients with a Germline BRCA-Mutated Advanced Breast Cancer: Detailed Safety Analyses from the Phase III EMBRACA Trial. The oncologist. 2019. Epub 2019/11/27. doi: 10.1634/theoncologist.2019-0493 . 32162822 PMC7066700

[11] Haddad G, Saade MC, Eid R, Haddad FG, Kourie HR. PARP inhibitors: a tsunami of indications in different malignancies. Pharmacogenomics. 2020;21(3):221–30. Epub 2020/01/23. doi: 10.2217/pgs-2019-0113 . 31967513

[12] IQWIG. Talazoparib (Mammakarzinom)- Nutzenbewertung gemäß §35a SGB V 2020 [11.07.2020]. Available from: https://www.iqwig.de/download/a20-48_talazoparib_nutzenbewertung-35a-sgb-v_v1-0.pdf.

[13] Bundesausschuss G. Beschluss des Gemeinsamen Bundesausschusses über eine Änderung der Arzneimittel-Richtlinie (AM-RL): Anlage XII- Nutzenbewertung von Arzneimitteln mit neuen Wirkstoffen nach §35a SGB V Talazoparib (Mammakarzinom, BRCA1/2-Mutation, HER2-) [05.07.2022]. Available from: https://www.g-ba.de/beschluesse/4547/.

[14] Schwabe UP, Dieter; Ludwig, Wolf-Dieter; Klauber, Jürgen. Arzneimittelverordnungsreport 2019: Pressekonferenz der Arzneimittelkommission der deutschen Ärzteschaft (AkdÄ) und des Wissenschaftlichen Instituts der AOK (WIdO) 24. September 2019, Berlin; [11.07.2020]. Available from: https://www.wido.de/fileadmin/Dateien/Dokumente/Publikationen_Produkte/Buchreihen/Arzneiverordnungsreport/wido_arz_pk_0919_avr_2019.pdf.

[15] Luengo-Fernandez R, Leal J, Gray A, Sullivan R. Economic burden of cancer across the European Union: a population-based cost analysis. Lancet Oncology. 2013;14(12):1165–74. Epub 2013/10/18. doi: 10.1016/S1470-2045(13)70442-X . 24131614

[16] Vogler S, Paris V, Ferrario A, Wirtz VJ, de Joncheere K, Schneider P, et al. How Can Pricing and Reimbursement Policies Improve Affordable Access to Medicines? Lessons Learned from European Countries. Applied health economics and health policy. 2017;15(3):307–21. Epub 2017/01/08. doi: 10.1007/s40258-016-0300-z . 28063134

[17] Light DW, Kantarjian H. Market spiral pricing of cancer drugs. Cancer. 2013;119(22):3900–2. Epub 2013/09/05. doi: 10.1002/cncr.28321 . 24002792

[18] Grandt DS, Ingrid. BARMER GEK Arzneimittelreport. Schriftenreihe zur Gesundheitsanalyse Band 3 2017 [11.07.2017]. Available from: https://www.barmer.de/presse/infothek/studien-und-reports/arzneimittelreporte/arzneimittelreport2017-121728.

[19] Wieseler B, McGauran N, Kaiser T. New drugs: where did we go wrong and what can we do better? BMJ (Clinical research edition). 2019;366:l4340. Epub 2019/07/12. doi: 10.1136/bmj.l4340 . 31292109

[20] Guy H, Walder L, Fisher M. Cost-Effectiveness of Niraparib Versus Routine Surveillance, Olaparib and Rucaparib for the Maintenance Treatment of Patients with Ovarian Cancer in the United States. PharmacoEconomics. 2019;37(3):391–405. Epub 2018/11/28. doi: 10.1007/s40273-018-0745-z ; PubMed Central PMCID: PMC6386009. 30478649 PMC6386009

[21] Smith HJ, Walters Haygood CL, Arend RC, Leath CA 3rd, Straughn JM Jr., PARP inhibitor maintenance therapy for patients with platinum-sensitive recurrent ovarian cancer: a cost-effectiveness analysis. Gynecologic Oncology. 2015;139(1):59–62. Epub 2015/08/26. doi: 10.1016/j.ygyno.2015.08.013 . 26303225

[22] Wolford JE, Bai J, Moore KN, Kristeleit R, Monk BJ, Tewari KS. Cost-effectiveness of niraparib, rucaparib, and olaparib for treatment of platinum-resistant, recurrent ovarian carcinoma. Gynecologic Oncology. 2020. Epub 2020/03/17. doi: 10.1016/j.ygyno.2020.02.030 . 32173049 PMC7410501

[23] Zhong L, Tran AT, Tomasino T, Nugent E, Smith JA. Cost-Effectiveness of Niraparib and Olaparib as Maintenance Therapy for Patients with Platinum-Sensitive Recurrent Ovarian Cancer. Journal of managed care & specialty pharmacy. 2018;24(12):1219–28. Epub 2018/11/28. doi: 10.18553/jmcp.2018.24.12.1219 . 30479195 PMC10397875

[24] Olry de Labry Lima A, Špacírová Z, Fénix-Caballero S, Hoces AM, Vegas AS, Aranzana MC, et al. Cost-utility of talazoparib monotherapy treatment for locally advanced or metastatic breast cancer in Spain. Breast). 2021;58:27–33. Epub 2021/04/26. doi: 10.1016/j.breast.2021.04.004 . 33895483 PMC8099594

[25] Eisenhauer EA, Therasse P, Bogaerts J, Schwartz LH, Sargent D, Ford R, et al. New response evaluation criteria in solid tumours: revised RECIST guideline (version 1.1). European journal of cancer (Oxford, England: 1990). 2009;45(2):228–47. Epub 2008/12/23. doi: 10.1016/j.ejca.2008.10.026 . 19097774

[26] Sonnenberg FA, Beck JR. Markov models in medical decision making: a practical guide. Medical Decision Making. 1993;13(4):322–38. Epub 1993/10/01. doi: 10.1177/0272989X9301300409 . 8246705

[27] Waschke A, Arefian H, Walter J, Hartmann M, Maschmann J, Kalff R. Cost-effectiveness of the long-term use of temozolomide for treating newly diagnosed glioblastoma in Germany. Journal of Neuro-oncology. 2018;138(2):359–67. Epub 2018/02/23. doi: 10.1007/s11060-018-2804-x . 29468446

[28] Ishak KJ, Kreif N, Benedict A, Muszbek N. Overview of parametric survival analysis for health-economic applications. PharmacoEconomics. 2013;31(8):663–75. Epub 2013/05/16. doi: 10.1007/s40273-013-0064-3 . 23673905

[29] Latimer NR. Survival analysis for economic evaluations alongside clinical trials—extrapolation with patient-level data: inconsistencies, limitations, and a practical guide. Medical Decision Making. 2013;33(6):743–54. Epub 2013/01/24. doi: 10.1177/0272989x12472398 . 23341049

[30] IQWIG. Allgemeine Methoden. Version 6 [11.07.2020]. Available from: https://www.iqwig.de/de/methoden/methodenpapier.3020.html.

[31] Peasgood T, Ward SE, Brazier J. Health-state utility values in breast cancer. Expert review of pharmacoeconomics & outcomes research. 2010;10(5):553–66. Epub 2010/10/19. doi: 10.1586/erp.10.65 . 20950071

[32] Paracha N, Thuresson PO, Moreno SG, MacGilchrist KS. Health state utility values in locally advanced and metastatic breast cancer by treatment line: a systematic review. Expert review of pharmacoeconomics & outcomes research. 2016;16(5):549–59. Epub 2016/08/31. doi: 10.1080/14737167.2016.1222907 . 27574879

[33] Aaronson NK, Ahmedzai S, Bergman B, Bullinger M, Cull A, Duez NJ, et al. The European Organization for Research and Treatment of Cancer QLQ-C30: a quality-of-life instrument for use in international clinical trials in oncology. Journal of the National Cancer Institute. 1993;85(5):365–76. Epub 1993/03/03. doi: 10.1093/jnci/85.5.365 . 8433390

[34] Sherrill B, Amonkar MM, Stein S, Walker M, Geyer C, Cameron D. Q-TWiST analysis of lapatinib combined with capecitabine for the treatment of metastatic breast cancer. British journal of cancer. 2008;99(5):711–5. Epub 2008/08/30. doi: 10.1038/sj.bjc.6604501 ; PubMed Central PMCID: PMC2528149. 18728660 PMC2528149

[35] Cameron D, Casey M, Press M, Lindquist D, Pienkowski T, Romieu CG, et al. A phase III randomized comparison of lapatinib plus capecitabine versus capecitabine alone in women with advanced breast cancer that has progressed on trastuzumab: updated efficacy and biomarker analyses. Breast cancer research and treatment. 2008;112(3):533–43. Epub 2008/01/12. doi: 10.1007/s10549-007-9885-0 . 18188694

[36] Einheitlicher Bewertungsmaßstab (EBM): Stand Viertes Quartal 2019 2019 [11.07.2020]. Available from: https://www.kbv.de/media/sp/EBM_Gesamt_-_Stand_4._Quartal_2019.pdf.

[37] Reimbursement info app [11.07.2020]. Available from: https://app.reimbursement.info/drgs.

[38] Bionity.com—Körperoberfläche [11.07.2020]. Available from: https://www.bionity.com/de/lexikon/K%C3%B6rperoberfl%C3%A4che.html.

[39] Common Terminology of Adverse Events: National Cancer Institute; [19.02.2022]. Available from: https://ctep.cancer.gov/protocoldevelopment/electronic_applications/ctc.htm#ctc_60.

[40] Onkologie Leitlinienprogramm (Deutsche Krebsgesellschaft, Deutsche Krebshilfe, AWMF): S3-Leitlinie Früherkennung, Diagnose, Therapie und Nachsorge des Mammakarzinoms, Version 4.3, 2020 [cited 11.07.2020]. Available from: https://www.leitlinienprogramm-onkologie.de/fileadmin/user_upload/Downloads/Leitlinien/Mammakarzinom_4_0/Version_4.3/LL_Mammakarzinom_Langversion_4.3.pdf.

[41] Wu B, Miao Y, Bai Y, Ye M, Xu Y, Chen H, et al. Subgroup economic analysis for glioblastoma in a health resource-limited setting. PloS one. 2012;7(4):e34588. Epub 2012/04/19. doi: 10.1371/journal.pone.0034588 ; PubMed Central PMCID: PMC3325281. 22511951 PMC3325281

[42] Mamiya H, Tahara RK, Tolaney SM, Choudhry NK, Najafzadeh M. Cost-effectiveness of palbociclib in hormone receptor-positive advanced breast cancer. Annals of Oncology. 2017;28(8):1825–31. Epub 2017/05/05. doi: 10.1093/annonc/mdx201 . 28472324

[43] Durkee BY, Qian Y, Pollom EL, King MT, Dudley SA, Shaffer JL, et al. Cost-Effectiveness of Pertuzumab in Human Epidermal Growth Factor Receptor 2-Positive Metastatic Breast Cancer. Journal of Clinical Oncology. 2016;34(9):902–9. Epub 2015/09/10. doi: 10.1200/JCO.2015.62.9105 ; PubMed Central PMCID: PMC5070553 online at www.jco.org. Author contributions are found at the end of this article. 26351332 PMC5070553

[44] Gordon N, Stemmer SM, Greenberg D, Goldstein DA. Trajectories of Injectable Cancer Drug Costs After Launch in the United States. Journal of Clinical Oncology. 2018;36(4):319–25. Epub 2017/10/11. doi: 10.1200/JCO.2016.72.2124 . 29016226

[45] Litton JK, Scoggins ME, Hess KR, Adrada BE, Murthy RK, Damodaran S, et al. Neoadjuvant Talazoparib for Patients With Operable Breast Cancer With a Germline BRCA Pathogenic Variant. Journal of Clinical Oncology. 2020;38(5):388–94. Epub 2019/08/29. doi: 10.1200/JCO.19.01304 ; PubMed Central PMCID: PMC7351336. 31461380 PMC7351336

