# Correction: Cost-effectiveness of talazoparib for patients with locally advanced or metastasized breast cancer in Germany

**DOI:** 10.1371/journal.pone.0303776

**Published:** 2024-05-09

**Authors:** Florian Schwarz, Habibollah Arefian, Michael Hartmann, Ingo Runnebaum

There is an error in affiliation 5 for author Ingo Runnebaum. The correct affiliation 5 is: Cancer Center Central Germany, Jena, Germany.
